# Outcome-volume relationships and transhiatal esophagectomy: minimizing “failure to rescue”

**DOI:** 10.1186/s13022-014-0009-3

**Published:** 2014-12-19

**Authors:** Renee L Arlow, Dirk F Moore, Chunxia Chen, John Langenfeld, David A August

**Affiliations:** Department of Surgery, Division of Surgical Oncology, Rutgers Cancer Institute of New Jersey, 195 Little Albany Street, New Brunswick, New Jersey 08903-2601 USA; Department of Biostatistics, Rutgers Cancer Institute of New Jersey, 195 Little Albany Street, New Brunswick, New Jersey 08903-2601 USA; Department of Surgery, Section of Thoracic Surgery, Rutgers Robert Wood Johnson Medical School and The Rutgers Cancer Institute of New Jersey, 195 Little Albany Street, New Brunswick, New Jersey 08903-2601 USA

**Keywords:** Esophagectomy, Esophageal cancer, Failure to rescue, Outcome-volume relationships, Quality improvement, Postoperative complication

## Abstract

**Background:**

The objective of this study is to describe the system and technical factors that enabled our moderate size transhiatal esophagectomy program to achieve low mortality rates.

**Methods:**

A retrospective chart review was conducted on 200 consecutive patients who underwent transhiatal esophagectomy at Robert Wood Johnson University Hospital. Primary outcomes included operative times, estimated blood loss, frequency and nature of complications, and lengths of stay in the hospital and the intensive care unit.

**Results:**

In general, surgical outcomes tended to improve over the course of this study. We identified decreased operative time, intra-operative blood loss, frequency of complications, and lengths of intensive care unit and hospital stay as the program matured. Through coordinated actions of the surgical and anesthesia teams, all intraoperative injuries were responded to in an effective, emergent fashion and all but one patient was saved. This resulted in an inhospital and 30-day mortality rate of only 0.5%.

**Conclusions:**

Our study suggests that a dual attending approach, focus on avoiding “failure to rescue”, increased volume, and a surgeon driven commitment to quality improvement may lead to low mortality rates after transhiatal esophagectomy.

## Background

The concept of “failure to rescue”, first defined by Silber et al, refers to the inability to prevent patient mortality following a major complication [1]. Although esophagectomy requires exacting intra-operative technique, it is still attended by complication rates reported to be as high as 25-50%, even at experienced centers [2-5]. This emphasizes the importance of avoiding “failure to rescue” and the potentially devastating consequences associated with esophagectomy [6]. Outcome-volume relationships have been described for many surgical procedures, including esophagectomy [7-17]. While obtaining favorable outcomes is dependent on both hospital and surgeon volume, moderate volume programs can achieve good outcomes by avoiding the devastating consequences of “failure to rescue”.

This manuscript explores the evolution of one institution’s esophagectomy program to determine the factors that contributed to a low inhospital and 30-day mortality rate of 0.5%, despite being only a moderate volume center. Complications, care structures, and technical factors are analyzed to determine how the program performed over time and to determine how key elements such as a dual attending approach, focus on avoiding “failure to rescue”, and a surgeon driven commitment to quality improvement affected outcomes. The lessons learned may provide insight for those attempting to develop successful intermediate volume complex surgery programs.

## Methods

A retrospective chart review was conducted on 212 consecutive patients taken to the operating room for attempted transhiatal esophagectomy between February, 2000 and April, 2013 at Robert Wood Johnson University Hospital. All cases were performed jointly by one of two thoracic surgeons and one surgical oncologist. The second thoracic surgeon performed only 16 cases and data analyzed with and without her cases revealed no statistically significant differences. Therefore, all cases were analyzed together. Patients were identified from a prospectively maintained secure database. All hospital charts were obtained from medical records and reviewed by the same two investigators independently of one another. The primary outcomes were prospectively determined before data collection was started. These outcomes included estimated blood loss and intraoperative complications derived from the operative reports. The frequency and nature of postoperative complications and the length of stay in the intensive care unit (ICU) were determined based on daily physician progress notes. The hospital length of stay was determined by admission and discharge date and operative times were determined based on anesthesia records.

**Figure 1:**
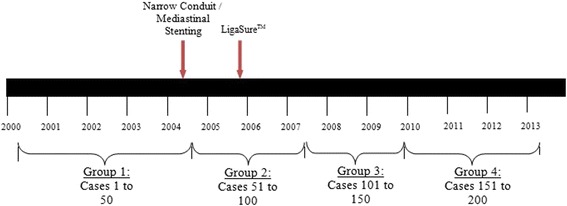
**Timeline for the introduction of technical changes.**

The cohort of completed esophagectomies was sequentially divided into 4 groups to assess the overall influence of surgeon and hospital experience on outcomes: Group 1 (cases 1-50), Group 2 (cases 51-100), Group 3 (cases 101-150), and Group 4 (cases 151-200) (twelve cases were excluded because the esophagectomy was not performed) (Figure 1). Surgeon driven technical changes that evolved over time were analyzed to assess their impact upon outcomes, including use of the LigaSure device, mediastinal stenting and creation of a narrow conduit. Institutional review board approval for this study was granted by the Rutgers Robert Wood Johnson Medical School IRB.

### Operative technique

All patients underwent esophagectomy by an open transhiatal technique with gastric mobilization and feeding jejunostomy. The abdominal cavity was entered through a midline incision and careful exploration was undertaken to rule out metastatic disease. After surrounding the distal esophagus with a Penrose drain, the greater curvature of the stomach was mobilized, taking a wide berth around the gastroepiploic arcade to preserve the blood supply to the gastric conduit. At the outset of this study, gastric mobilization was performed by dividing the greater curvature vessels (including the short gastric vessels) and ligating them manually. Beginning in November 2005, the LigaSure device was introduced for mobilization, and this was a discrete technical change decided upon by the attending surgeons. The thoracic portion of the dissection was initially performed using surgical clips and/or electrocautery for hemostasis, but beginning in 2005 the LigaSure device was also introduced.

Initially gastrointestinal continuity was re-established by attempting to maximize gastric conduit width. In June 2004, a change in operative technique was initiated to facilitate pull-up of the gastric conduit through the posterior mediastinum. The conduit width was narrowed from 10 cm to approximately 6 cm. Simultaneously, to facilitate passage of the conduit through the posterior mediastinum from the abdomen to the left neck, a pair of Jackson-Pratt drains were threaded through the posterior mediastinum to elevate the mediastinal structures. In all cases, the gastroesophageal anastomosis was fashioned using a side-to-side functional end-to-end stapled technique and this technique remained consistent throughout the study period [18].

### Statistical analysis

Statistical analysis was performed in SPSS Statistical Software (version 18) using analysis of variance for continuous variables, the Chi-squared test for categorical variables and the Wilcoxon rank sum test for ordinal variables. Linear regression was utilized to assess the influence of surgeon experience and technical changes on the number of admissions to the intensive care unit. Differences that achieved a two-tailed p-value less than 0.05 were considered statistically significant.

## Results

From February 2000 to April 2013, transhiatal esophagectomy was attempted in 212 patients at Robert Wood Johnson University Hospital. In twelve patients the operation was aborted because of the discovery of unsuspected metastatic disease in the liver or omentum and these cases were excluded from analysis. In two additional cases esophagectomy was not completed due to injury to either the gastroepiploic artery or the thoracic aorta. Transhiatal esophagectomy was completed in all but six of the remaining cases; two were converted to Ivor-Lewis esophagectomy, three to a “3-hole esophagectomy” including right thoracotomy, and one to a total gastrectomy with Roux-en-Y esophagojejunostomy.

**Figure 2:**
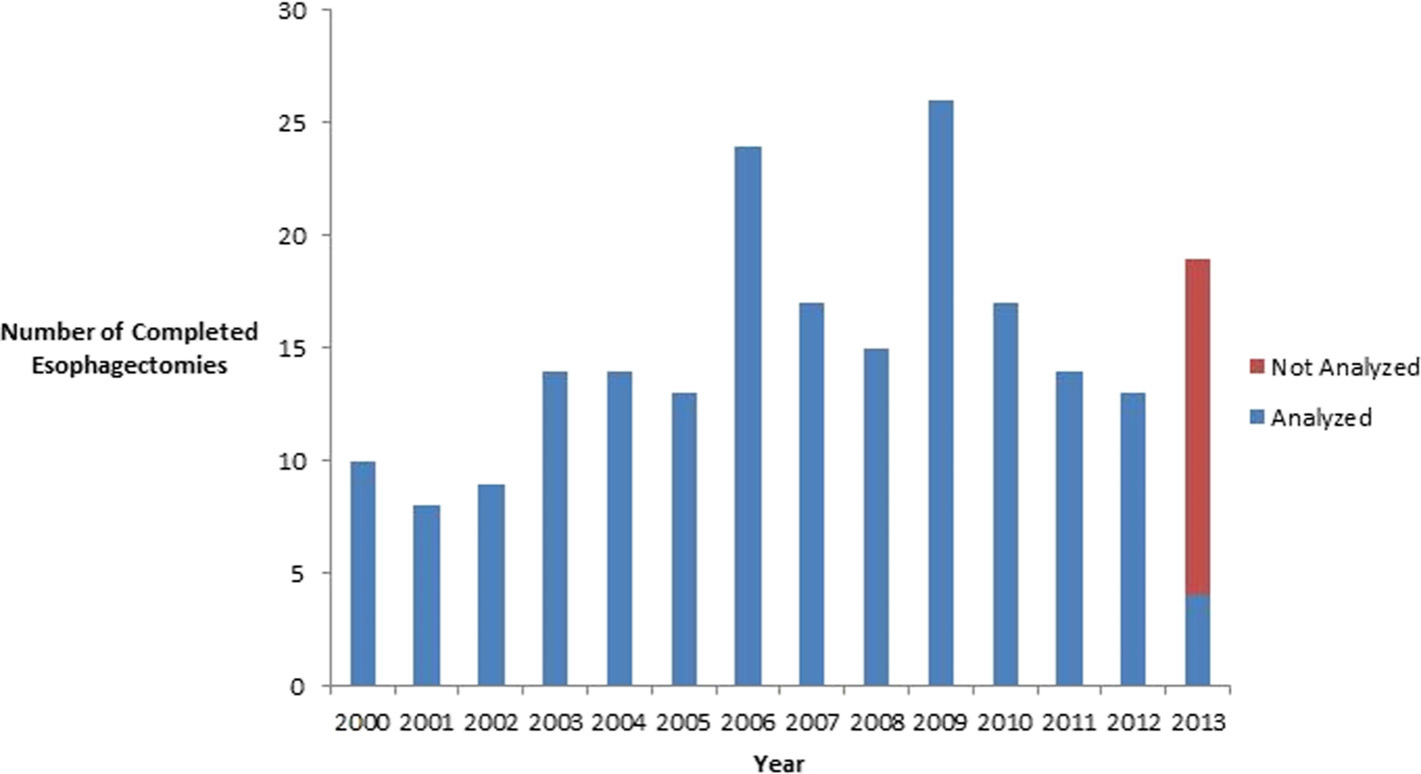
**Number of esophagectomies completed per year.**

Over time, the number of annual cases gradually increased (Figure 2). By 2006, the program surpassed the target number of 15 esophagectomies per year to be considered a high volume center and remained at this volume for most of the remaining years [8]. The median age at the time of operation was 62 years and surgery was performed for invasive malignancy in 91% of cases. Baseline characteristics are summarized in Table 1.

**Table 1:** **Patient demographics and pre**-**operative characteristics**

**Variable**	**All cases**	**Group 1**	**Group 2**	**Group 3**	**Group 4**	**P**-**value**
Male: Female	5:1	6:1	5:1	4:1	9:1	0.41
Median age at operation, years (range)	62 (40-82)	62 (46-82)	62 (40-77)	62 (40-80)	64 (46-80)	0.54
Smoking history (%)	74	82	78	72	62	0.12
Body Mass Index (kg/m^2^)	27.6	27.9	26.9	28.4	27.3	0.63
Mean pre-operative albumin (g/dL)	3.7	3.5	3.7	3.7	3.7	0.054
1 or more comorbidities (%)^†^	62	60	60	56	70	0.52
Histology (%)^‡^						0.06
Adenocarcinoma	60	82	80	74	74	
Squamous cell carcinoma	16	10	10	14	13	
High grade dysplasia / CIS	18	6	4	8	9	
Other	2	2	2	0	2	
Pre-Operative Stage (%)^§^						0.40
0	0	2	0	0	0	
I	18	2	26	20	24	
II	11	4	6	12	22	
III	27	10	34	34	28	
IV	3	0	2	4	6	
Unknown	41	82	32	30	20	
Post-Operative Stage (%)						0.51
0	26	20	22	22	23	
I	22	40	16	26	26	
II	24	18	32	22	24	
III	28	20	22	22	23	
IV	0	0	2	0	1	
Neoadjuvant therapy (%)	60	40	56	68	74	0.003*

*CIS* – carcinoma in situ.

Group 1 = cases 1 – 50.

Group 2 = cases 51 – 100.

Group 3 = cases 101 – 150.

Group 4 = cases 151-200.

^†^Patients who met the following criteria were considered to have a comorbid condition: a diagnosis of diabetes mellitus, chronic obstructive pulmonary disease, peripheral vascular disease or stroke; a diagnosis of hypertension or current treatment with an anti-hypertensive medication; a diagnosis of coronary artery disease or history of coronary artery stenting or coronary artery bypass surgery; and a history of previous deep vein thrombosis or pulmonary embolism.

^‡^For histology, other includes: gastrointestinal stromal tumor, melanoma, and poorly differentiated carcinoma with neuroendocrine features.

^§^All esophagectomy patients who were considered stage IV preoperatively had celiac lymph node disease on endoscopic ultrasound (n = 6). Patients were reported to have unknown preoperative staging if the endoscopic ultrasound report was missing from their hospital chart.

*p < 0.05

### Complications

During the course of this study only one inhospital or 30-day mortality occurred. This event was due to intraoperative exsanguination from an aortic injury during chest dissection. Overall, intraoperative complications occurred in 6.5% of cases and included: five splenic injuries requiring splenectomy, one hemiazygous vein, four aortic injuries, one right ventricular injury, one brief episode of asystole, and one intraoperative myocardial infarction. Of these 13 potentially life threatening complications, 6 of which required entry into the chest, all but 1 was successfully rescued.

One hundred and eighteen post-operative complications occurred (in 37% of patients), including anastomotic leak (14%), chyle leak (6%), recurrent laryngeal nerve paralysis (7%), ventilator dependent respiratory failure (15%), pneumonia or sepsis (13%), pulmonary embolism or deep venous thrombosis (4%), or myocardial infarction (2%) (Table 2). Anastomotic leaks were defined as clinical evidence of leak necessitating incision and drainage with evacuation of purulent material. None of the anastomotic leaks in this case series occurred before postoperative day 5 and only 4 of the 27 anastomotic leaks (15%) ultimately required reoperation. Of those reoperations, two were for drainage, one of which required diverting esophagostomy and two for repair of tracheoesophageal fistulas, one of which required emergent ligation of the internal jugular vein. Of the 11 patients who developed a chyle leak, only 3 (27%) required reoperation.

**Table 2:** **Number of cases with a post**-**operative complication**

**Complication**	**All cases**	**Group 1**	**Group 2**	**Group 3**	**Group 4**	**P-** **value**
Anastomotic leak	27 (14%)	10 (20%)	5 (10%)	5 (10%)	7 (14%)	0.43
Chyle leak	11 (6%)	1 (2%)	6 (12%)	3 (6%)	1 (2%)	0.09
Recurrent laryngeal nerve injury (temporary or permanent)	13 (7%)	4 (8%)	4 (8%)	2 (4%)	3 (6%)	0.82
Ventilator dependent respiratory failure	29 (15%)	8 (16%)	9 (18%)	8 (16%)	4 (8%)	0.51
Pneumonia or sepsis	25 (13%)	5 (10%)	9 (18%)	9 (18%)	2 (4%)	0.10
Pulmonary embolism or deep venous thrombosis	9 (4%)	5 (10%)	1 (2%)	1 (2%)	2 (4%)	0.18
Myocardial infarction	4 (2%)	0 (0%)	0 (0%)	3 (6%)	1 (2%)	0.11
1 or more major^†^	61 (31%)	18 (36%)	17 (35%)	14 (28%)	12 (24%)	0.56

Group 1 = cases 1 – 50.

Group 2 = cases 51 – 100.

Group 3 = cases 101 – 150.

Group 4 = cases 151 – 200.

^†^1 or more major complication(s) include at least one of the following: anastomotic leak, chyle leak, ventilator dependent respiratory failure and recurrent laryngeal nerve injury.

The most common cause of morbidity in our study was respiratory failure (15%), which was defined as any patient who required reintubation. Interestingly, in almost all cases, respiratory failure was associated with another complication, including recurrent laryngeal nerve injury, anastomotic leak, chyle leak, pneumonia or sepsis. In fact, 22 of the 29 patients (76%) with respiratory failure had one or more of the previously mentioned complications. This may support harboring a high index of suspicion for additional complications in any patient who develops respiratory failure.

The mortality rate in this cohort was 0.5% and there were no postoperative mortalities. A review of the patients who experienced life threatening complications was performed to identify system based elements that may have resulted in a high rate of successful rescue. Of the 13 intraoperative complications, 11 involved life threatening hemorrhage. Eight were recognized intraoperatively (3 aortic bleeds, 3 splenic bleeds, and one patient each with hemiazygous and right ventricular injuries). Through coordinated actions of the surgical and anesthesia teams, all of these injuries were responded to in an effective, emergent fashion and all but one patient were saved. Of the remaining cases, one aortic bleed was recognized within 45 minutes post-operatively, and an emergent return to the operating room resulted in successful rescue. In another case, recognition of an intraoperative myocardial infarction resulted in emergent cardiac catheterization with successful thrombectomy of the right coronary artery. This complication was identified because a three minute episode of hypotension accompanied by transient ST elevations intraoperatively was recognized to be at an inappropriate time in the case (i.e. the surgeon was not in the mediastinum and there was no excessive bleeding or other source to explain the hypotension) prompting further work-up. Similarly, one splenic hemorrhage that presented on post-operative day six was emergently returned to the operating room with successful rescue of the patient. Overall, there was only one intraoperative mortality and none of the 61 patients who experienced major complications, including ventilator dependent respiratory failure, chyle leak, anastomotic leak, or nerve injury experienced inhospital or 30-day mortality [19].

### Change over time

Since the study period spanned twelve years, a cohort analysis was performed to determine if any changes occurred as a result of either increased surgeon experience or specific surgeon driven changes in technique, including use of the narrow conduit/mediastinal stenting and the LigaSure device. Overall, although the rate of most major postoperative complications tended to decrease during the study period, none reached statistical significance (Table 2). This also held true for operating room time and median estimated blood loss (Table 3).

**Table 3:** **Univariate analysis of perioperative and postoperative outcomes**

**Group**	**Mean OR time (** **hours)**	**Median estimated blood loss** **(cc)** **a**	**Percent of cases with an intraoperative complication**	**Percent of cases with one or more major postoperative complication** **(s)**	**Median ICU length of stay**, **days** ** (range)**	**Median hospital length of stay**, **days** **(range)**	**Percent of patients requiring any ICU stay**	**Percentage of patients discharged to home**
All Cases	5.5	500	7	31	0 (0-89)	8.5 (5-107)	41	76
Group 1^†^	5.7	600	8	36	2 (0-89)	9 (6-89)	80	80
Group 2^‡^	5.6	500	4	35	0 (0-27)	9 (6-46)	27	84
Group 3^§^	5.5	450	6	28	0 (0-52)	8.5 (5-107)	34	70
Group 4^¶^	5.3	400	8	24	0 (0-18)	8 (6-30)	22	68
**P value**	**0.27**	**0.77**	**0.82**	**0.56**	**0.17**	**0.22**	0.000*	**0.18**
Wide conduit	5.8	600	7	35	2 (0-89)	9 (6-89)	80	80
Narrow conduit	5.5	450	6	30	0 (0-52)	8 (5-107)	29	74
**P value**	**0.12**	**0.56**	**0.99**	**0.51**	**0.14**	**0.29**	0.000*	**0.39**
Before LigaSure	5.7	600	9	37	2 (0-89)	9 (6-89)	73	83
After LigaSure	5.4	450	5	28	0 (0-52)	8 (5-107)	26	71
**P value**	**0.08**	**0.46**	**0.26**	**0.24**	**0.1**	**0.18**	0.000*	**0.06**

*EBL* – estimated blood loss; *ICU* – intensive care unit; *OR* – operating room.

^†^Group 1 = cases 1 – 50.

^‡^Group 2 = cases 51 – 100.

^§^Group 3 = cases 101 – 150.

^¶^Group 4 = cases 151 – 200.

*p < 0.05.

### Intensive care unit (ICU) and hospital lengths of stay and discharge disposition

Patients were admitted postoperatively to a monitored bed on the surgical oncology floor unless clinical circumstances dictated otherwise. Reasons for ICU stay included atrial fibrillation, postoperative myocardial infarction, and respiratory failure. Fifteen of the eighteen cases requiring ICU stay greater than ten days were due to prolonged respiratory failure. The length of a patient’s ICU and hospital stay were influenced by both surgeon experience as well as technical changes (Table 3). This was statistically significant with regards to patients who required any ICU stay, decreasing from 80 in the first cohort to 22 percent in last (Table 3). Based on multivariate analysis, only the creation of a narrow conduit/mediastinal stenting was associated with a statistically significant decreased need for admission to the ICU (Table 4). This, however, was independent of major postoperative complications and therefore likely had more to do with a change in surgeon practice towards less routine admittance to the ICU than improved outcomes.

**Table 4:** **Multivariate analysis of influence of factors on number of patient requiring any intensive care unit** (**ICU**) **stay**

**Dependent Variable**	**Independent variable(s)**	**P-** **value**
Number of patients requiring any ICU stay	Chronologic Group	0.95
	Narrow conduit/Mediastinal Stenting	0.045*
	LigaSure	0.06

*ICU* – intensive care unit.

* – statistically significant P-value.

Additionally, we examined whether patient discharge disposition (home vs care facility) changed over time. We wanted to discern whether more patients were being discharged to skilled nursing facilities instead of home as hospital and ICU lengths of stay decreased. In fact the number of patients discharged home as opposed to care facilities did decline over time, although this was not statistically significant (Table 3).

## Discussion

Despite numerous studies supporting the outcome-volume relationship, centralization of complex procedures to high volume institutions is not uniformly advantageous. Volume based referral alone can set a dangerous precedent because it is based on the assumption that volume is a direct proxy for quality care, and this is unlikely to be uniformly true [20]. In fact, a review of hospitals in Washington state found significant variation in quality, even among hospitals that met the Leapfrog standards for a high volume hospital (≥13 esophagectomies per year) [21].

This study reviewed a single institution’s transhiatal esophagectomy program to demonstrate that a moderate volume program can achieve low mortality rates. In fact, the mortality rate in our case series (0.5%) compared quite favorably to the national average of 7% as reported by Kohn *et al*. based on the Nationwide Inpatient Sample database from 1998–2006. The Nationwide Inpatient Sample database is the largest all-payer inpatient care databases in the United States, including about eight million patients each year [22]. Additionally, Merkow *et al*. reported a 30-day mortality rate of 2.5% for transhiatal esophagectomy based on 164 hospitals participating in the ACS-NSQIP Program [23].

The mortality rate in our case series (0.5%) is similar even to very high volume programs that perform more than 40-50 cases per year. For example, a systematic review and meta-analysis from 2000-2011 by Markar *et al*. revealed an overall 30-day mortality rate of 0.73% at such very high volume centers [24]. Interestingly, the major complication rate (31%) at our institution is comparable to national averages suggesting that the low mortality rate may be attributed to avoiding “failure to rescue” [6].

Prior to the study period, Robert Wood Johnson University Hospital was considered a low-volume esophagectomy program. All cases were performed solely by a thoracic surgeon and fewer than ten cases were performed per year. In 2000, a decision was made to perform all operations jointly by one surgical oncologist and one thoracic surgeon. We believe our program’s success was multifactorial and can be attributed both to increases in volume and to having two established attending surgeons with distinct areas of specialization accompanied by well-trained support staff. Intraoperatively, both surgeons worked simultaneously, each assisted by a separate general surgery resident and scrub nurse. This interaction helped facilitate management of complications as well as complex decision making.

Postoperatively, two experienced surgeons, in addition to their full resident teams, greatly increased the chance that subtle clinical findings were noticed and addressed. We have fostered a major emphasis on daily communication between all members of the team. Management disagreements are quickly adjudicated by the attending surgeons, generally in favor of pursuing a more aggressive diagnostic and therapeutic approach. It is likely that the low mortality rate in this series can be attributed both to improved volumes and to this approach through minimizing the devastating consequences of “failure to rescue” [6].

This program has instituted a commitment to analyze outcomes and to adjust operative technique and patient management accordingly. For example, the high incidence of anastomotic leaks during the first 50 cases (20%) served as an impetus to change operative technique. It was felt that difficulties bringing the gastric conduit up through the posterior mediastinum to the neck for anastomosis may have been compromising the stomach. This led to the decision to make the conduit narrower, and to temporarily enlarge the posterior mediastinum during the actual pull-up with drainage tubes placed under tension. Following this change in technique, the anastomotic leak rate fell from 20% to 10%. In addition, use of the LigaSure device was another discrete technical change decided upon by the attending surgeons in order to facilitate gastric mobilization and thoracic dissection. This lead to a decrease in median blood loss from 600 to 400 from groups 1 to 4, respectively. Although not statistically significant, operative time decreased by 25 minutes over the course of the study, suggesting that increased surgeon experience with the procedure (“practice makes perfect”) may also be relevant.

## Conclusions

Overall, while esophagectomy remains a technically demanding operation with high morbidity and mortality, good outcomes can be achieved at moderate volume centers. A dual attending approach in an academic center with specialized support staff and frequent communication among team members, as well as a strong commitment to both quality improvement and to maintaining adequate volume, may prevent “failure to rescue”. We hope that lessons learned may provide insight for those attempting to develop successful intermediate volume complex surgery programs.
